# Potential Role for Combined Subtype-Selective Targeting of M_1_ and M_3_ Muscarinic Receptors in Gastrointestinal and Liver Diseases

**DOI:** 10.3389/fphar.2021.786105

**Published:** 2021-11-04

**Authors:** Mazen Tolaymat, Margaret H. Sundel, Madeline Alizadeh, Guofeng Xie, Jean-Pierre Raufman

**Affiliations:** ^1^ Department of Medicine, Division of Gastroenterology and Hepatology, University of Maryland School of Medicine, Baltimore, MD, United States; ^2^ Department of Surgery, University of Maryland School of Medicine, Baltimore, MD, United States; ^3^ VA Maryland Healthcare System, Baltimore, MD, United States; ^4^ Marlene and Stewart Greenebaum Comprehensive Cancer Center, University of Maryland School of Medicine, Baltimore, MD, United States; ^5^ Department of Biochemistry and Molecular Biology, University of Maryland School of Medicine, Baltimore, MD, United States

**Keywords:** muscarinic receptors, G protein-coupled receptors, gastrointestinal physiology, gastrointestinal disease, liver disease, cancer

## Abstract

Despite structural similarity, the five subtypes comprising the cholinergic muscarinic family of G protein-coupled receptors regulate remarkably diverse biological functions. This mini review focuses on the closely related and commonly co-expressed M_1_R and M_3_R muscarinic acetylcholine receptor subtypes encoded respectively by *CHRM1* and *CHRM3*. Activated M_1_R and M_3_R signal via G_q_ and downstream initiate phospholipid turnover, changes in cell calcium levels, and activation of protein kinases that alter gene transcription and ultimately cell function. The unexpectedly divergent effects of M_1_R and M_3_R activation, despite similar receptor structure, distribution, and signaling, are puzzling. To explore this conundrum, we focus on the gastrointestinal (GI) tract and liver because abundant data identify opposing effects of M_1_R and M_3_R activation on the progression of gastric, pancreatic, and colon cancer, and liver injury and fibrosis. Whereas M_3_R activation promotes GI neoplasia, M_1_R activation appears protective. In contrast, in murine liver injury models, M_3_R activation promotes and M_1_R activation mitigates liver fibrosis. We analyze these findings critically, consider their therapeutic implications, and review the pharmacology and availability for research and therapeutics of M_1_R and M_3_R-selective agonists and antagonists. We conclude by considering gaps in knowledge and other factors that hinder the application of these drugs and the development of new agents to treat GI and liver diseases.

## Introduction

Muscarinic receptors (MRs) are class A (Rhodopsin-like) guanine nucleotide protein-coupled receptors (GPCRs) differentiated from other cholinergic receptors by preferential binding of muscarine rather than nicotine (Eglen, 2012; Tiwari et al., 2013). MRs are further subcategorized into five subtypes, designated M_1_R through M_5_R and encoded by *CHRM1*-*CHRM5*, each of which modulates a range of parasympathetic activities (Caulfield and Birdsall, 1998). These functionalities depend on tissue and membrane localization (Koenig and Edwardson, 1996; Nathanson, 2008). Like other GPCRs, MRs are characterized by seven transmembrane helices designated TM1 through TM7, forming a partially-spiral configuration within the cell membrane (Hulme et al., 2003). Acetylcholine (ACh) binds on the extracellular aspect of MRs in a pocket formed by TM3, TM6, and TM7 residues. The five MR subtypes share 82–92% transmembrane region homology, with 64–82% sequence similarity overall (Maeda et al., 2019). As GPCRs, activated MRs interact with heterotrimeric guanine nucleotide-binding proteins (G-proteins), classified by their *α* subunits, to activate downstream targets.

Although classically responsive to ACh, MRs, like other GPCRs, possess allosteric binding sites for naturally occurring and engineered non-ACh ligands, with varying degrees of preference; allosteric effects may result in surprising downstream actions in cell types not previously considered responsive to muscarinic signaling (Tolaymat et al., 2019). ACh and these “non-traditional” ligands provide MRs with the ability to modulate a broad repertoire of cells and biological systems including those associated with neuronal signaling, immune function, and cell trafficking, proliferation, and differentiation (Wessler and Kirkpatrick, 2008; McLean et al., 2016). Dysregulated post-MR signaling is associated with unregulated cell proliferation and cancer progression (Chen et al., 2019), an “overactive” bladder (Abrams et al., 2006), autoimmune diseases (Berg et al., 2010; Lee et al., 2013) and psychiatric disorders (Scarr, 2012; Vakalopoulos, 2014; Jeon et al., 2015). In addition to the discovery that non-traditional ligands can modify MR function, the production and release of ACh is more widespread than originally thought; a wide variety of non-neuronal cells express choline acetyltransferase (ChAT), the key enzyme needed to convert acetyl CoA and choline into ACh (Wessler and Kirkpatrick, 2012). Colon cancers, for example, express high levels of ChAT (Cheng et al., 2008). The variety of processes modulated by MRs has invited extensive research into the potential use of agonists, antagonists, and allosteric modulators for myriad disorders.

### Muscarinic Receptor Distribution and Post-Receptor Signaling

MRs are expressed by a wide variety of tissues and cell types and control key digestive and metabolic functions. Salivary gland secretion, gastric, and intestinal fluid transport, cell proliferation, mucus production, motility, and mesenteric vascular constriction and dilation are all responsive to MR signaling (Tobin et al., 2009; Muise et al., 2017). In the stomach, M_3_R, M_4_R, and M_5_R activation modulates hydrochloric acid secretion from parietal cells (Aihara et al., 2005), and M_1_R and M_3_R activation stimulates pepsinogen secretion from chief cells (Xie et al., 2005). GI motility, through intestinal smooth muscle cell action, involves communication between the central and enteric nervous systems. These effects are partially mediated by M_1_R through M_3_R (Moro et al., 2005), with M_2_R and M_3_R playing a role in regulating longitudinal muscle contraction, and all three MR subtypes involved in circular muscle function (Harrington et al., 2010; Tanahashi et al., 2021). MR-mediated regulation of smooth muscle function extends throughout the entire GI tract. Nonetheless, it is likely that MRs play additional roles in regulating small intestinal function; for example, M_2_R is expressed in the stem cell compartment and may be involved in enterocyte turnover (Muise et al., 2017). ACh has both pro- (Koyama et al., 1992; Brunn et al., 1995) and anti-inflammatory (Pavlov and Tracey, 2006) effects, the latter mediated in part by reducing systemic levels of tumor necrosis factor. While the ubiquity of MRs within the digestive tract makes them attractive therapeutic targets to modulate health and disease, this same ubiquity complicates efforts to design selective agents while minimizing off-target adverse effects.

Responses of MRs to ligand binding are subtype specific. Activation of odd-numbered MRs (M_1_R, M_3_R, and M_5_R) stimulates phospholipid turnover and increases intracellular calcium levels while activation of even-numbered MRs (M_2_R, M_4_R) inhibits adenylyl cyclase activity, thereby reducing levels of intracellular cAMP. M_1_R, M_3_R, and M_5_R (MR_odd_) canonically couple to G_q/11_ which induces the phospholipase C-mediated hydrolysis of phosphatidylinositol (4.5)-bisphosphate into diacylglycerol and inositol (1,4,5)-trisphosphate. The latter binds an endoplasmic reticulum receptor stimulating intracellular calcium release. However, these may represent oversimplifications; experimental findings suggest differential interactions of individual MR_odd_ and MR_even_ with their downstream targets. For example, although both M_1_R and M_3_R signal through phospholipase C, CHO cells expressing M_1_R exhibited four-fold greater cAMP production in response to carbachol compared to cells expressing M_3_R (Burford et al., 1995). Likewise, although M_2_R and M_4_R (MR_even_) act primarily by binding G_i/o_ family proteins to alter adenylyl cyclase activity, their actions can also prolong potassium channel opening, thereby causing cellular hyperpolarization (Bubser et al., 2012).

These general principles do not tell the whole story–despite substantial sequence homology among MR subtypes they demonstrate surprising individuality in their responses to stimuli, even within the same cell and when responding to the same ligand. Pancreatic acinar cells provide a useful model to study muscarinic control of exocrine digestive function. Using acinar cells prepared from M_1_R- and M_3_R-deficient mice as well as M_1_/M_3_ chimeric receptors, Nakamura et al., demonstrated greater ACh-induced IP_3_ release in cells expressing only M_1_R compared to those expressing uniquely M_3_R (Nakamura et al., 2013). Moreover, in M_3_R-compared to M_1_R-expressing cells, these differences were associated respectively with oscillatory versus monotonic patterns of cytosolic calcium release. Oscillatory calcium release was a function of a C-terminal region of M_3_R with considerable variability among MR subtypes (Nakamura et al., 2013). In murine gastric chief cells, both M_1_R and M_3_R mediate pepsinogen secretion–deletion of either MR subtype reduces and combined M_1_R and M_3_R deficiency ablates cholinergic agonist-induced proenzyme secretion (Xie et al., 2005). Thus, in some cell types, MR_odd_ have overlapping functions whereas in other cell types, MR subtype signaling appears divergent. In addition to the influence of their cell and tissue localization, other mechanistic differences between MR subtypes result in sometimes-opposing effects. GPCRs, including MRs, can also undergo “pre-coupling”, wherein a stable multimeric complex is present before ligand binding. Unlike other MR subtypes, M_1_R and M_3_R pre-couple with G_i/o_ G-proteins, their non-preferential G protein, thereby potentially altering downstream effects (Jakubík et al., 2011). Crystal structures of inactive M_1-4_R subtypes provide some insight into different allosteric and orthosteric binding sites (Kruse et al., 2012; Thal et al., 2016), but our understanding of the resulting functional differences between MR subtypes continues to evolve.

### Effects of Dysregulated M_1_R and M_3_R Signaling on Non-Proliferative Disorders Involving the Digestive System

As a result of their central role in maintaining homeostasis in the GI tract, dysregulated MR signaling can be an important modifier of intestinal disease. In Hirschsprung disease, lack of mucosal cholinergic innervation in aganglionic colon segments increases the risk of postoperative enterocolitis (Keck et al., 2021). In diarrhea-predominant irritable bowel syndrome (IBS-D) without a concomitant psychiatric disorder, pyridostigmine (an acetylcholinesterase inhibitor) induces a stronger IL-6 response that is highly correlated with symptoms (Dinan et al., 2008). Given the pharmacotherapies targeting MRs already approved or being explored to treat IBS (Tables 1, 2), achieving a more precise mechanistic understanding of the role MR dysregulation plays in IBS is important.

**TABLE 1:** FDA/EMA approved muscarinic receptor antagonists and agonists.

Generic (Trade) name	Activity/MR selectivity	Dose range/Route	Approved indications
Benztropine (Cogentin) Bolden et al. (1992)	M_1_R Ant	0.5–6 mg/day IM/IV/PO	Parkinson’s disease, extrapyramidal symptoms, dystonia
Biperiden (Akineton) Eltze and Figala (1988)	M_1_R Ant	1–16 mg/day PO, 2.5–5 mg IM/IV	Parkinson’s disease, extrapyramidal symptoms
Dicyclomine (Bentyl) Giachetti et al. (1986)	M_1_R Ant	20–160 mg/day PO	Irritable bowel syndrome
Pirenzipine (Gastrozepin) Bolden et al. (1992)	M_1_R Ant	100–150 mg/day PO	Peptic ulcer disease
Trihexyphenidyl (Artane) Giachetti et al. (1986)	M_1_R Ant	5–15 mg/day PO	Parkinson’s disease
Cevimeline/AF-102B (Evoxac) Weber and Keating (2008b)	M_1_R, M_3_R Agonist	90 mg/day PO	Xerostomia in Sjogren’s syndrome
Oxybutynin (Ditropan) Andersson and Chapple (2001)	M_1_R, M_3_R Ant	5–30 mg/day PO; topical and transdermal	Overactive bladder
Aclidinium (Tudorza Pressair) Beier et al. (2013)	M_3_R Ant	800 mcg/daily inhaled	Chronic obstructive pulmonary disease
Darifenacin (Enablex) Yamada et al. (2006)	M_3_R Ant	7.5–15 mg/day PO	Overactive bladder
Solifenacin (VESIcare) Oki et al. (2005)	M_3_R Ant	5–10 mg/day PO	Overactive bladder
Aceclidine* (Glaunorm) Erickson and Schroeder (2000)	NS Agonist	Topical	Glaucoma
Bethanechol (Urecholine)	NS Agonist	30–200 mg/day PO	Urinary retention
Methacholine	NS Agonist	1–380 mcg	Bronchial airway hyperactivity
Pilocarpine (Salagen, Isopto Carpine) Zimmerman (1981)	NS Agonist	15–30 mg/day PO	Xerostomia, glaucoma
Atropine (Atropen)	NS Ant	0.5–3 mg IV/IM; available as inhalant	Bradycardia, inhibit secretions; mushroom/organophosphate poisoning
Scopolamine (Transderm-Scop)	NS Ant	1.5 mg skin patch; available PO, IM, IV	Nausea, sedation, GI and genitourinary spasm
Tolterodine (Detrol) Hills et al. (1998)	NS Ant	2–4 mg/day PO	Overactive bladder

Ant, antagonist; EMA, European Medicines Agency; FDA, United States Food and Drug Administration; IM, intramuscular; IV, intravenous; NS, nonselective; PO, oral. *, not FDA approved.

**TABLE 2:** Selective M_1_R/M_3_R agents used for research and under clinical investigation.

Agent	Activity/MR selectivity	Source	Potential clinical applications
2′ biaryl amides Budzik et al. (2010)	M_1_R Agonist	GlaxoSmithKline	
77-LH-28-1 Langmead et al. (2008)	M_1_R Agonist		Alzheimer’s disease, schizophrenia
AC-42 Heinrich et al. (2009)	M_1_R Agonist		
HTL0018318 Bakker et al. (2021)	M_1_R Agonist	Sosei Heptares Therapeutics	Dementia
PPBI Wood et al. (2017)	M_1_R Agonist	AstraZeneca	Analgesia
Nitrocaramiphen Hudkins et al. (1993)	M_1_R Ant		
PIPE-307	M_1_R Ant	Pipeline Therapeutics	Multiple sclerosis; clinical trials (NCT04941781, NCT04725175)
PIPE-359 Schrader et al. (2021)	M_1_R Ant	Pipeline Therapeutics	
Telenzepine Eveleigh et al. (1989)	M_1_R Ant	Theracos	Peptic ulcer disease; obesity (Clinical trial NCT01155531)
VU 0255035 Tsentsevitsky et al. (2017)	M_1_R Ant	Vanderbilt University	Seizure disorder
L-689,660 Hargreaves et al., (1992)	M_1_R, M_3_R Agonist		
Oxotremorine Veena et al., (2011)	M_1_R, M_3_R Agonist		
R2HBJJ Hua et al. (2012)	M_1_R, M_3_R Ant		Non-small cell lung cancer
McN-A-343 Mitchelson (2012b)	M_1_R, M_4_R Agonist		
Xanomeline Heinrich et al. (2009)	M_1_R, M_4_R Agonist		Alzheimer’s disease
4-DAMP Honda et al. (2007)	M_3_R Ant		
AZD8871 Aparici et al. (2019)	M_3_R Ant	Almirall	Chronic obstructive lung disease
DA-8010 Lee et al. (2019)	M_3_R Ant		Overactive bladder
DAU 5884 Gosens et al. (2004)	M_3_R Ant		
J-104129 Mitsuya et al. (1999)	M_3_R Ant	Merck	Obstructive airway disease
Temiverine Kikukawa et al. (1998)	M_3_R Ant		Urinary incontinence
YM905 Kobayashi et al. (2001)	M_3_R Ant	Astellas (Yamanouchi)	Irritable bowel syndrome
Arecoline Heinrich et al. (2009)	NS Agonist		Alzheimer’s disease, schizophrenia

Ant, antagonist; NS, nonselective.

Diseases associated with MR dysregulation are not restricted to the lower GI tract. In the stomach, cholinergic signaling is balanced with histamine and gastrin release to regulate gastric acid levels; peptic ulcer disease is associated with greater MR expression in the gastric body, whereas progressive MR loss in that region is associated with chronic gastritis (Pfeiffer et al., 1995). In progressive systemic sclerosis and Sjogren’s syndrome, an autoimmune condition which impairs lacrimal and salivary function, esophageal dysmotility may be associated with anti-M_3_R antibodies (Goldblatt et al., 2002; Kawaguchi et al., 2009; Gyger and Baron, 2012); anti-M_3_R antibodies are also reported in progressive systemic sclerosis with anal dysmotility (Singh et al., 2009; Gyger and Baron, 2012). Intravenous immunoglobulin to neutralize anti-M_3_R antibodies may be beneficial (Smith et al., 2005).

Compared to the normal liver, individuals with primary biliary cholangitis (PBC) are more likely to have a *CHRM3* single nucleotide polymorphism (rs4620530) of uncertain significance; this is not associated with baseline disease characteristics or treatment responses (Greverath et al., 2020). PBC is more commonly associated with anti-M_3_R antibodies than other liver diseases (Tsuboi et al., 2014); those with anti-M_3_R antibodies are more likely to have a benign disease course. Nonetheless, M_3_R antibody levels do not correlate with treatment responses or serological markers either at baseline or during the disease course (Mayer et al., 2020). A subset of patients with PBC develop Sjogren’s syndrome; the shared increase in anti-M_3_R antibody levels in both conditions suggests overlapping features could form the basis for a mutual treatment.

### Divergent Effects of M_1_R and M_3_R Signaling on Digestive Tract Cell Proliferation and Neoplasia

MRs play key roles in normal cell proliferation and turnover. As reviewed by Campoy et al. (2016), presumably to benefit tumor progression, neoplastic cells hijack MR-dependent proliferative signal transduction pathways. Treating neoplastic cells with exogenous ACh and inhibiting ACh hydrolysis promotes their proliferation and, conversely, reducing M_3_R expression and activation is anti-proliferative. Moreover, because neoplastic cells tend to lose cellular polarity, receptors normally expressed on the basolateral membrane may be expressed more diffusely around the cell membrane, thereby facilitating their access to orthosteric and allosteric ligands in the tumor microenvironment and GI lumen (Cheng et al., 2002). For example, bile acids, at concentrations achieved in stool, promote atropine-inhibitable colon cancer cell proliferation (Cheng and Raufman, 2005).

Abundant data support the conclusion that M_3_R plays an important role in colon cancer progression. In mouse models of sporadic and genetic colon cancer, using azoxymethane (AOM)-treated and *Apc* ^*Min/+*^ mice, respectively, *Chrm3* ablation with resulting M_3_R deficiency substantially reduces the intestinal tumor burden (Raufman et al., 2008; Raufman et al., 2011). As M_3_R deficiency primarily reduces the number of adenocarcinomas rather than adenomas, the major impact of blocking M_3_R activation appears to be on promotion, rather than initiation, of neoplasia. M_3_R activation has similar pro-proliferative effects on gastric cancer (Hayakawa et al., 2017; Wang et al., 2018). M_3_R expression is enhanced in cholangiocarcinoma and associated with reduced cell differentiation, perineural invasion, and metastasis (Feng et al., 2012; Feng et al., 2018).

In contrast to the impact of M_3_R deficiency, M_1_R deficiency in mice does not attenuate, and may modestly enhance, AOM-induced colon carcinogenesis. Strikingly, mice with combined M_1_R and M_3_R deficiency develop as many colon tumors as control mice (Cheng et al., 2014); that is, M_1_R deficiency negates the anti-neoplastic effects of M_3_R deficiency. Likewise, M_1_R agonism appears protective against pancreatic ductal adenocarcinoma (PDAC) and counteracts enhanced carcinogenesis following vagotomy (Renz et al., 2018), suggesting a potential therapeutic opportunity. In contrast, in hepatocellular and prostate carcinomas, M_1_R activation promotes cellular migration and invasiveness (Yin et al., 2018; Zhang et al., 2020). Notably, many of these studies are limited by using global rather than conditional knockout mouse models. Hence, it remains uncertain whether the respective MR deficiencies are due to effects on neoplastic cells versus other cellular elements in the tumor microenvironment (e.g., immunocytes). Nonetheless, these observations argue strongly for the importance of MR subtype selectivity in designing and developing therapeutics.

Branches of the vagus nerve, a major source of ACh signaling within the GI tract, innervate the liver and modulate hepatocyte regeneration by progenitor cells and fibrosis by stellate cells (Cassiman et al., 2002). The current lack of effective anti-fibrotic therapies highlights the potential of leveraging these muscarinic actions to prevent or reverse fibrosis in advanced liver disease and stimulate hepatocyte regeneration. For example, in rodents, carbon tetrachloride (CCl_4_)-induced hepatic fibrosis can be attenuated by vagotomy and treatment with atropine (Lam et al., 2008). M_3_R expression and activation protects against AOM-induced liver fibrosis (Khurana et al., 2010; Khurana et al., 2013; Rachakonda et al., 2015). Surprisingly, M_1_R expression and activation appears to have opposite effects, worsening AOM-induced hepatic fibrosis (Rachakonda et al., 2015). Thus, in the absence of effective anti-fibrotic therapy, manipulation of MR subtype activity to limit or reverse fibrosis may have therapeutic potential although, again, divergent effects in different tissues warrants caution.

### Use of MR Agonists and Antagonists to Treat Digestive Tract Disease

MR subtype, tissue distribution, and off-target side effects have hindered efforts to manipulate MR activity precisely and effectively with drugs. MR antagonists are most effective in treating chronic obstructive pulmonary disease and overactive bladder (Table 1) (Eglen et al., 1999; Athanasopoulos and Giannitsas, 2011)—their utility for GI and hepatic disorders is currently limited. Cholinesterase inhibitors that increase ACh levels, also used clinically for digestive tract disorders, have similar limitations as their actions are largely non-selective. Adverse effects with these classes of drugs are attributed primarily to off-target effects on the CNS (e.g., convulsions, confusion) and other peripheral MR subtypes (e.g., sialorrhea, rhinitis, diaphoresis, diarrhea, nausea, vomiting, and bronchospasm). Novel MR agonists and antagonists are currently under investigation primarily for diseases of the central nervous system such as Alzheimer’s disease and schizophrenia (Table 2) (Davie et al., 2013).

Several MR agonists and cholinesterase inhibitors are in clinical use. Oral and topical pilocarpine. and cevimeline (Evoxac), an M_3_R-selective activator, augment salivary gland secretions in xerostomia due to radiation therapy and Sjogren’s syndrome (Iga et al., 1998; Fife et al., 2002; Petrone et al., 2002; Weber and Keating, 2008a; Berk, 2008; Mitchelson, 2012a; Davies and Thompson, 2015; Panarese and Moshirfar, 2021). Bethanechol, a structural analogue of ACh that resists hydrolysis by cholinesterases, has potential to treat esophageal dysmotility. Currently approved to treat urinary retention and neurogenic bladder (Gaitonde et al., 2019), bethanechol strengthens esophageal contractions in subjects with ineffective esophageal motility (Agrawal et al., 2007) and augments lower esophageal sphincter pressure in gastroesophageal reflux disease (Farrell et al., 1973). Nonetheless, in a pilot study, topical bethanechol did not significantly improve esophageal motility (O’Rourke et al., 2013). Edrophonium, a cholinesterase inhibitor used to diagnose myasthenia gravis, was used to provoke esophageal spasm in the investigation of non-cardiac chest pain, but the lack of correlation between symptoms and objective changes in esophageal manometry limited its utility (Botoman, 2002).

Gastric acid secretion is controlled by a mix of cholinergic muscarinic stimulation and hormonal signaling by gastrin and histamine; thus, only partial inhibition of acid release is achieved with anti-muscarinic agents. Consequently, histamine-2 receptor and H^+^-K^+^ATPase (proton pump) inhibitors are highly successful and MR antagonists rarely prescribed. Pirenzepine (Gastrozepin), an M_1_R antagonist that is not FDA approved, has limited use to treat acid-related disorders in the EU (Tryba and Cook, 1997). Scopolamine, a non-selective MR antagonist, is commonly used as a transdermal patch for nausea associated with anesthesia or motion sickness (Riad and Hithe, 2021).

Dicyclomine (Bentyl), an M_1_R- and M_3_R-selective antagonist that inhibits small and large intestinal motility, is used as an anti-spasmodic agent to treat IBS (Giachetti et al., 1986; Doods et al., 1987). Neostigmine, a cholinesterase inhibitor, is used to treat acute intestinal pseudo-obstruction associated with critical illness or opioid use, another condition of impaired smooth muscle motility. Colonic decompression may be achieved with intravenous neostigmine (De Giorgio et al., 2001), although cardiac monitoring is important and rapid administration of atropine may be required for resulting bradycardia.

Although a potential role for modulating MR activity to treat cancer was demonstrated in a variety of cell types (Shah et al., 2009), except for an ongoing trial to investigate the utility of bethanechol before surgery for resectable PDAC (U.S. National Library of Medicine, 2021), the efficacy of modulators of MR activity in digestive tract cancers has not been tested in the clinic. Moreover, anti-tumor efficacy may be limited by the inability to achieve adequate concentrations in target tissues while, at the same time, preventing off-target adverse effects. An ideal agent would exhibit target organ and MR-subtype specificity, goals hampered by the extensive similarity between orthosteric and allosteric ligand binding sites among the five MR subtypes (Liu et al., 2018). Studies of naturally occurring ligands, such as muscarinic toxins in snake venom, have provided insight into how subtype-selective agents may be formulated (Maeda et al., 2020). Such agents with potential for oncotherapy continue to be developed. For example, the M_3_R-specific antagonist darifenacin which is approved to treat bladder dysfunction (Yamada et al., 2006) reportedly inhibits tumor progression and invasiveness in human-derived cell lines, most recently in colorectal cancer cell lines (Hering et al., 2021). As darifenacin is in clinical use with a known safety profile, it is an attractive candidate for adjunctive therapy, especially for cancers already shown to overexpress M_3_R, like colon cancer cells (Frucht et al., 1999; Cheng et al., 2014), PDAC (Zhang et al., 2016), and non-small cell lung cancer (Lin et al., 2014).

Some therapeutic approaches may circumvent the need for MR subtype and tissue specificity. For example, treating colorectal cancers with poorly absorbed oral agents or drugs with extensive first-pass metabolism may target GI mucosal lesions with limited systemic side effects. However, even within a limited area of distribution, MRs are not constrained to only one downstream signaling pathway; the same receptor may have contradictory effects on neighboring cell types. Even when occupying the same binding pocket, ligands can influence the activation of pathways on other cell membrane surfaces via signaling bias and functional selectivity (Randáková and Jakubík, 2021). A ligand may bind several MR subtypes, but only activate one or a few, thereby compensating for binding pocket homogeneity. Furthermore, through selective interactions with residues in the binding pocket of a single subtype, ligands can encourage activated receptor configurations that favor interaction with certain G proteins. As an example, the MR agonist cevimeline increased intracellular calcium levels in Chinese hamster ovary (CHO) cells transfected with rat M_1_R but did not increase cAMP levels. In contrast, carbachol, a non-selective MR agonist, elevated both calcium and cAMP levels (Gurwitz et al., 1994). Even more intriguing, cevimeline did not activate signaling in M_3_R-transfected cells, contrary to its clinical use in Sjogren’s syndrome which is thought to be mediated by M_3_R activation. This complexity makes it difficult to predict the clinical effects of new MR agonists and antagonists but suggests highly selective agents can be developed.

## Conclusion: Current Gaps in Knowledge, Drug Development, and Therapeutic Opportunities

MR activation via the vagus nerve, the longest and most complex cranial nerve, and within the enteric nervous system, is a major modifier of normal and pathological GI and hepatic function. As reviewed here, MRs and the machinery needed to produce their ligands are not limited to neuronal cells. Abundant evidence exists that “non-traditional” ligands (e.g., other than ACh) mediate paracrine and autocrine signaling by orthosteric and allosteric interactions with MR subtypes. These findings highlight the potential for treating a broad range of physiological and disease processes with MR subtype-selective agents. Numerous non-selective and subtype-selective orthosteric ligands that modify MR signaling have been developed and investigated to treat a variety of digestive diseases (Tables 1, 2); allosteric regulation of MR activity represents a presently untapped reservoir of agents that can be designed or repurposed to alter cell function. Overall, there has been limited clinical use of both orthosteric and allosteric modifiers of MR function. Despite more than 20 years of evidence supporting an important role for MR activation in GI cancer progression, currently only one clinical trial is investigating the efficacy of a drug to modulate MR activity as adjunctive treatment for a digestive tract cancer, PDAC (ClinicalTrials.gov Identifier: NCT03572283).

Extensive sequence homology between the five MR subtypes hampers efforts to create agents with sufficiently selective actions and, thereby, limited off-target toxicity. Adding to this complexity is the observation that a receptor subtype on one cell type may activate different downstream signaling pathways, depending on the interaction between ligand and receptor and the conformational changes instigated by this interaction. In addition to subtype-specificity, ideal agents must possess sufficient tissue specificity to prevent deleterious action on neighboring and distant tissues. In this regard, targeting diseases involving intestinal mucosa, e.g., neoplasia, may be advantaged by developing agents with limited GI absorption or extensive first-pass metabolism. Current gaps in knowledge include a better understanding of subtype-selective allosteric modulation of MR function, an area in its infancy.

Lastly, several observations reviewed above suggest great potential for leveraging the divergent actions of M_1_R and M_3_R activation to treat GI cancers. Thus, a drug design challenge is to develop a molecule with dual functionality as an M_1_R agonist and M_3_R antagonist. Moreover, it has not escaped our attention that developing an agent with the opposite properties may be useful to prevent or reverse hepatic fibrosis. Success at creating dual agonists for different bile acid receptors in the gut suggests that although the challenge is formidable, it can be overcome (Ito et al., 2021). As our understanding of these complex signaling mechanisms evolves and the medicinal chemistry needed to develop MR subtype-specific agents progresses, targeting MR subtypes is likely to become a valuable adjunct for treating a variety of digestive tract disorders, including cancer.
